# Post-Market Surveillance of Predictive Decision Support Tools in the Presence of Confounding Medical Interventions using Causal Inference

**DOI:** 10.21203/rs.3.rs-9918108/v1

**Published:** 2026-06-22

**Authors:** Sardar Ansari, Brittany Baur, Andrew Admon

**Affiliations:** 1 Department of Emergency Medicine, University of Michigan Medical School, 1540 E Hospital Dr, Ann Arbor, 48109, MI, US.; 2 Department of Internal Medicine, University of Michigan Medical School, 1500 E Medical Center Dr, Ann Arbor, 48109, MI, US.; 3 Medicine Service, LTC Charles S. Kettles VA Medical Center, 2215 Fuller Rd, Ann Arbor, 48105, MI, US.; 4 The Max Harry Weil Institute for Critical Care Research and Innovation, University of Michigan, 2800 Plymouth Rd, Ann Arbor, 48109, MI, US.

**Keywords:** Post-Market Surveillance, Artificial Intelligence, Machine Learning, Performance Degradation, Data Drift, Model Drift

## Abstract

Predictive AI models have been widely adopted in various clinical applications. While there has been tremendous focus on the pre-deployment performance assessment of these models, there has been little attention paid to their post-deployment evaluation. It has previously been shown that post-market surveillance of predictive AI models can be complicated by confounding medical interventions (CMI), actions that clinicians take in response to the model predictions to prevent the predicted adverse outcomes. This impacts the labels that the model will be evaluated on, leading to biased performance estimates. The more successful the model is in preventing adverse outcomes, the lower its performance is going to appear. To address this issue, we propose a novel approach to monitor the performance of deployed predictive models using causal inference techniques. We provide simulation results demonstrating that the proposed approach can provide unbiased estimates of all performance metrics for models with binary target variable.

## 1 Introduction

Monitoring the performance of predictive artificial intelligence decision support tools (AI-DSTs) after deployment is challenging because many such models prompt clinical interventions that prevent the outcomes they predict [1]. For example, the objective of a recent predictive model for HIV infection is to prompt treatment with pre-exposure prophylaxis, reducing the risk of HIV infection [2]. If the interventions prompted by a model are successful, the target labels used for model evaluation will differ after the model is deployed. These confounding medical interventions (CMIs) cause models to appear ‘incorrect’ upon later validation, preventing effective degradation detection [3]. In fact, bias in the observed performance estimates increases as the model becomes more successful in improving clinical outcomes.

One solution to address CMIs is to withhold model outputs for randomly selected patients, validating model performance among this subset. This amounts to a perpetual or recurring randomized controlled trial. While this enables accurate ongoing performance estimation, it raises ethical concerns as clinicians adopt an AI-DST into their workflows and its use becomes the standard of care. The absence of AI-DST output may then put patients at risk or unfairly reduce the clinical resources allocated to them. A second proposed solution involves monitoring clinical outcomes as a surrogate for AI-DST performance. While an accurate model paired with an effective intervention can improve clinical outcomes, a lack of observed improvements after AI-DST deployment is not necessarily due to poor model performance. It may instead reflect low clinician trust and adopt the AI-DST, or a lack of effective interventions for model-targeted conditions. Observed improvements in outcomes may also be due to seasonal changes or other shifts in clinical practice.

A third potential solution is to include “clinician intervention” as a model term [4]. This approach, which is akin to “adjusting for” the effects of clinician interventions, assumes that subjects receiving and not receiving treatment are *exchangeable* conditional on other model covariates. For this assumption to be valid, the datagenerating process—including all potential confounders of the treatment–outcome relationship—must be faithfully replicated in the prediction model, and there must be no unmeasured confounders or introduced selection bias. This is rarely true for published prediction models, which are often developed with goals that differ from causal models that estimate unbiased treatment effects. In fact, including clinician interventions as a predictor can exacerbate the problem because the intervention variable often contributes positively to model-predicted risks in non-causal models, resulting in an even larger discrepancy between the model output and the observed labels it will be evaluated against.

**Fig. 1:**
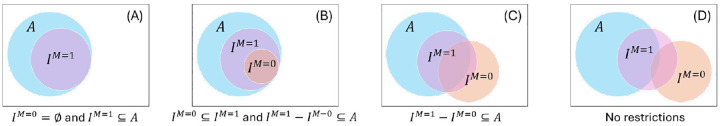
Scenarios in which patients who receive positive model predictions, denoted by A, receive interventions after the model is deployed, in increasing complexity of model degradation detection (left to right). Pre- and post-deployment interventions are denoted by $I^{M=0}$ and $I^{M=1}$, respectively, using counterfactual notation. **(A)** A new intervention is designed and deployed alongside the model where a subset of patients with a positive prediction receive the treatment (no pre-deployment interventions). **(B)** Model promotes an existing intervention. All patients who receive intervention pre-deployment also receive it post-deployment. **(C)** Same as (B) but some patients who received an intervention pre-deployment do not receive it post-deployment. **(D)** Same as (C) but some patients who did not receive an intervention pre-deployment and did not have an alert receive an intervention postdeployment due to introduction of other promoters of intervention such as changes in the standard of care, deployment of other coexisting AI-DSTs or new biomarkers.

Feng et al. proposed a score-based cumulative sum (CUSUM) procedure in a causal framework to identify model calibration changes after deployment [5]. Yet, their simulation results suggest that the CUSUM procedure’s effectiveness drops as clinician trust in the model increases. We will demonstrate that this is consequential because the bias introduced by CMIs worsens as trust increases (Section 2.1). Feng et al. also proposed a CUSUM procedure to monitor positive and negative predictive values [6]. This addresses scenarios where the intervention that ensues from the model alerts was not in use before model deployment (Figure 1A). However, it does not apply in more general scenarios (Figures 1B–1D), including when a model’s goal is to increase the use of treatments already in use (e.g., allocating existing interventions to patients at high risk of some adverse outcome). Also, both approaches provide a test to determine if a change in model performance has occurred but do not provide the magnitude of change or quantitative estimates of model performance.

We propose an approach using causal modeling to account for CMIs in the estimation of model performance and enable post-market monitoring of predictive AI-DSTs. A key to this approach is to separately consider (a) the effects of model recommendations on clinician decisions and (b) the effects of those decisions on predicted outcomes. If a model’s degraded performance over time is driven by clinicians acting on model predictions to avert adverse outcomes, validating models against counterfactual outcomes—outcomes that would have occurred had a clinician not intervened—can enable consistent post-deployment validation despite effective interventions that convert positive labels to negative. Table 1 summarizes solutions from the literature compared to our proposed solution.

## 2 Results

### 2.1 Effect of Confounding Medical Interventions on Post-Deployment Monitoring of a Real AI-DST

To demonstrate the effect of CMIs on observed model performance, simulation were conducted on a published EHR model [7–9] by introducing additional interventions. When a patient did not have an intervention in the data but was predicted as positive by the model, their intervention label was changed from 0 to 1 with probability $p_{\text{int}}$, referred to as *intervention frequency*. This simulates clinicians treating patients who were not treated otherwise as a result of the model predictions. For patients who experienced an adverse outcome in the data and their intervention label changed from 0 to 1 in the simulation, the adverse outcome label was changed from 1 to 0 with probability $p_{\text{eff}}$, referred to as *intervention effectiveness*. This simulates the additional interventions being effective in preventing the adverse outcome.

**Fig. 2:**
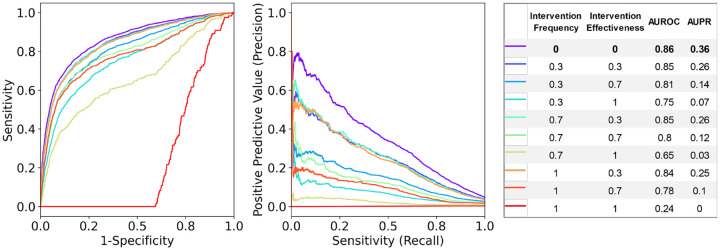
Receiver operating characteristic (ROC) and precision-recall (PR) curves for a previously published model [7–9]. Intervention frequency refers to the added probability of an intervention among patients who receive a model alert. Intervention effectiveness is the probability that the intervention prevents the adverse outcome. The original AUROC (0.86) and AUPR (0.36) correspond to the true model performance. Each other curve corresponds to simulated ROC and PR curves under varying post-deployment intervention frequencies and effectiveness rates. The AUROC decreases from 0.86 to 0.24 and AUPR decreases from 0.36 to 0.00 as the intervention frequency and effectiveness increase. Thus, bias in the observed AUROC and AUPR are greater as intervention frequency and effectiveness increase, despite true model performance remaining unchanged.

The model was then evaluated against the revised labels at different levels of $p_{\text{int}}$ and $p_{\text{eff}}$ (Figure 2). The results demonstrate that the area under the receiver operating curve (AUROC) and the area under the precision-recall curve (AUPR) drop significantly as intervention frequency and effectiveness approach 1.0, despite the fact that the true model predictive performance has not changed. If interpreted naively, this artifactual decay in performance may lead to counterproductive actions like updating or even abandoning effective models—each at a detriment to clinical outcomes. These results have been previously presented elsewhere [1].

### 2.2 Estimation of Model Performance using Causal Inference

**Fig. 3:**
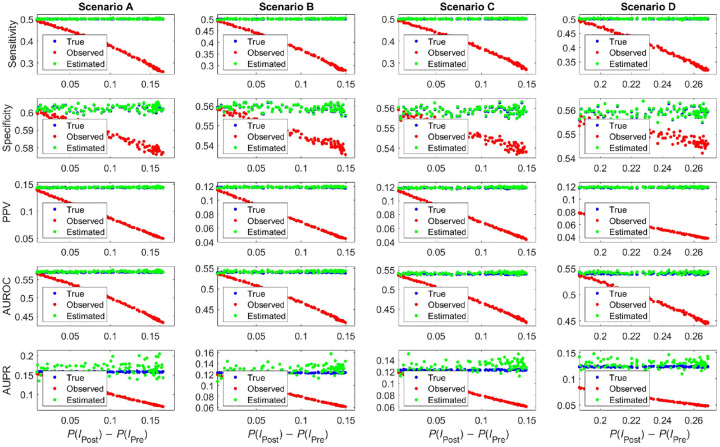
The simulation results showing the true, observed and estimated (using the equations in Table 2) performance metrics including sensitivity, specificity, PPV, AUROC and AUPR for the four scenarios depicted in Figure 1. The x axes correspond to the change in the intervention rate from pre- to post-deployment setting.

In Section 4, we derive equations for sensitivity, specificity and positive predictive value (PPV) of a predictive AI-DST after it has been deployed and true labels are masked by CMIs. We also demonstrate that NPV is not impacted by CMIs. The equations to calculate these performance metrics are summarized in Table 2. The calculation of sensitivity, specificity and PPV requires estimation of the causal effect of the AI-DST on the outcome that it predicts, CE(M). In Section 4.1, we demonstrate that

(1) $$\hat{CE}(M)=\hat{CE}\left(I|I_{\text{Post}}\right)P\left(I_{\text{Post}}\right)-\hat{CE}\left(I|I_{\text{Pre}}\right)P\left(I_{\text{Pre}}\right)$$

where CE(I) is the causal effect of the intervention that results from the model predictions on the predicted outcome and $I_{\text{Pre}}$ and $I_{\text{Post}}$ are the subsets of patients who receive the intervention before and after the AI-DST is deployed. Simulations were conducted to demonstrate the accuracy and effectiveness of this approach, as described in Section 4.4. simulation results are shown in Figure 3. The AI-DST did not experience drift and its performance remained constant as intervention frequency increased. This is depicted by the blue dots (often covered by the green dots) being invariant as interventions increase in Figure 3.

In Scenarios A-C, the increase in the event rate is entirely caused by the AI-DST, while in Scenario D, it is caused both by the AI-DST and another promoter of intervention such as a new biomarker that was introduced after the AI-DST was trained and tested. As such, the observed patterns in the true, observed and estimated metrics are similar in Scenarios A-C. However, the PPV and AUPR demonstrate different patterns in Scenario D with the bias always present even when the AI-DST is not effective in increasing the intervention rate. This is due to the presence of the new intervention promoter that creates a gap between the true and observed PPV and AUPR. Note that $P\left(I_{\text{Post}}\right)-P\left(I_{\text{Pre}}\right)$ is also always larger than 0 unlike Scenario A-C.

All observed performance metrics experience significant bias as the AI-DST becomes more effective in preventing the adverse outcome. For example, the observed sensitivity and PPV have approximately 40% and 65% relative bias, respectively, when the probability of intervention increases by 0.16. On the other hand, the estimated sensitivity, specificity and PPV closely track their true values in all four scenarios. This demonstrates that the equations in Table 2 are effective in correcting the bias introduced by CMIs. AUPR estimates demonstrated the highest variation. This is caused by P(A) in the denominator of the PPV equation. At high thresholds, A approaches an empty set, resulting in high variance in the estimation of P(A) and inflated ratios. This issue can potentially be addressed by clipping the PPV values when there are too few observation in A to generated accurate estimates.

## 3 Discussions

In this paper, we highlight the challenges associated with post-deployment monitoring of AI-DSTs due to the presence of CMIs that mask the true labels that the model should be evaluated on. We propose a solution to address this problem by separating the effect of the model on the clinical intervention from the effect of the intervention on the outcome that the model predicts using causal inference and counterfactual reasoning. We consider four different scenarios increasing in complexity, covering different ways the model can impact the interventions. The scenarios range from the introduction of a new intervention that did not exist before model deployment to simultaneous introduction of the AI-DST and another promoter of intervention such as a new predictive model or biomarker, both targeting an existing intervention aimed at preventing the adverse outcome.

The simulation results demonstrate that the proposed approach is highly effective in recovering all performance metrics including sensitivity, specificity, PPV, AUROC and AUPR, despite severe bias in the observed values of these metrics. We also demonstrate that our approach is effective in recovering the true ROC and PR curves while the observed curves are highly skewed by the CMIs. This is important to allow the model owners to adjust the model thresholds and obtain desired levels of performance and alarm rate when a drift is detected that results in a shift in the model calibration.

The proposed solution has several limitations. First, it requires an accurate model of the causal effect of the intervention on the target adverse outcome. This may require qualitative work to develop a causal DAG to account for all potential confounding variables. Similar to any treatment effect model, the exchangeability assumption (no unmeasured confounding) is untestable, complicating the monitoring attempts. However, the estimation of the causal effect is independent of the model and often does not change over time. Hence, the causal effect needs to be estimated once at any stage of model deployment (before or after). The assumption of constant intervention effect itself may need to be evaluated over time, but this can be done using basic monitoring techniques since the causal effect is not impacted by the CMIs.

Second limitation is associated with the types of DSTs that can be monitored using the proposed approach. The application of these methods is limited to AI-DSTs that predict binary outcomes and support single predictions per patient or encounter. Finally, the proposed approach assumes that the model impacts clinician decision only through alerts (positive predictions). However, there may be several pathways for clinicians to receive model predictions in some settings, limiting the impact of the proposed methods.

Monitoring AI-DSTs with multinomial targets, continuous targets, sequential predictions and in settings where the model predictions impact clinical decisions in ways other than binary alerts are the subject of future research.

## 4 Methods

We use conventional potential outcome notation in what follows. The superscripts indicate potential outcomes. We use D and $\bar{D}$ to denote the presence and absence of the outcome, I and $\bar{I}$ to denote the presence and absence of the intervention, M and $\bar{M}$ to denote the presence and absence of the model, and A to denote the model alert (positive predictions). The notations are also summarized in Table 3.

### 4.1 Performance Estimation when AI-DST is the Only Source of CMI

Scenario C in Figure 1 represents a situation where the deployment of AI-DST impacts the outcomes through CMIs, but no other model or initiative that targets the same patients or interventions is introduced into the system after the AI-DST deployment, resulting in changes in the event rates. We will begin with expanding the probability of the outcome in the absence of the model as

(2) $$P\left(D^{M=0}\right)=P\left(D^{M=0}|A\right)P(A)+P\left(D^{M=0}|\bar{A}\right)P(\bar{A})$$

Assuming the model can only impact the outcomes through alerts, PPV in the absence of the model can be calculated as

(3) $$P\left(D^{M=0}|A\right)=\frac{P\left(D^{M=0}\right)-P\left(D^{M=0}|\bar{A}\right)P(\bar{A})}{P(A)}=\frac{P\left(D^{M=0}\right)-P(D|\bar{A})P(\bar{A})}{P(A)}$$

where D and A correspond to the post-deployment observed adverse outcomes and alerts, respectively. Therefore, all components of the PPV calculation are observed except $P\left(D^{M=0}\right)$. In addition, the sensitivity in the absence of the model can be calculated as

(4) $$P\left(A|D^{M=0}\right)=\frac{P\left(D^{M=0}|A\right)P(A)}{P\left(D^{M=0}\right)}=\frac{P\left(D^{M=0}\right)-P(D|\bar{A})P(\bar{A})}{P\left(D^{M=0}\right)}=1-\frac{P(D|\bar{A})P(\bar{A})}{P\left(D^{M=0}\right)}$$

which also depends on observed quantities and $P\left(D^{M=0}\right)$. Likewise, the specificity can be estimated as

(5) $$P\left(\bar{A}|\bar{D^{M=0}}\right)=\frac{P\left(\bar{D^{M=0}}|\bar{A}\right)P(\bar{A})}{P\left(\bar{D^{M=0}}\right)}=\frac{P(\bar{A})-P\left(D^{M=0}|\bar{A}\right)P(\bar{A})}{1-P\left(D^{M=0}\right)}=\frac{P(\bar{A})-P(D|\bar{A})P(\bar{A})}{1-P\left(D^{M=0}\right)}$$

which also depends only on observed quantities and $P\left(D^{M=0}\right)$. The negative predictive value (NPV) can be calculated as

(6) $$P\left(\bar{D^{M=0}}|\bar{A}\right)=1-P\left(D^{M=0}|\bar{A}\right)=1-P(D|\bar{A})$$

which only depends on the observed probability of adverse outcome among patients without an alert. The last step holds because the assumption that the model impacts the interventions only through its alerts implies $P\left(D^{M=0}|\bar{A}\right)=P\left(D^{M=1}|\bar{A}\right)$. Therefore, calculation of NPV is not impacted by CMIs.

We can immediately see that unbiased estimates of PPV, sensitivity and specificity can be obtained by estimating $P\left(D^{M=0}\right)$ from the retrospective data if one assumes that there has been no change in the system (e.g., patient population and characteristics, clinician behavior, intervention effectiveness) besides deployment of the AI-DST. This is an untestable assumption since the counterfactual labels $D^{M=0}$ are systematically masked by the model predictions. In addition, this assumption is often violated due to the highly dynamic nature of the healthcare environment, evolving medical science and technology, and advancements in patient care. Under this assumption, the observed PPV, sensitivity and specificity can be easily corrected using equations 3, 4 and 5.

Since the assumption of static $D^{M=0}$ rarely holds, one needs to estimate $P\left(D^{M=0}\right)$ in order to measure the PPV, sensitivity and specificity of the model in the absence of CMIs. Since the probability of the outcome in the presence of the model, $P\left(D^{M=1}\right)$, is observed, we can alternatively estimate the causal effect (CE) of the model, $CE(M)=P\left(D^{M=1}\right)-P\left(D^{M=0}\right)=P(D)-P\left(D^{M=0}\right)$. The CE can be expanded as

(7) $$CE\left(M\right)=P\left(D^{M=1}\right)-P\left(D^{M=0}\right)=P\left(D^{M=1}|\bar{I^{M=0}}\bar{I^{M=1}}\right)P\left(\bar{I^{M=0}}\bar{I^{M=1}}\right)-P\left(D^{M=0}|\bar{I^{M=0}}\bar{I^{M=1}}\right)P\left(\bar{I^{M=0}}\bar{I^{M=1}}\right)+P\left(D^{M=1}|\bar{I^{M=0}}I^{M=1}\right)P\left(\bar{I^{M=0}}I^{M=1}\right)-P\left(D^{M=0}|\bar{I^{M=0}}I^{M=1}\right)P\left(\bar{I^{M=0}}I^{M=1}\right)+P\left(D^{M=1}|I^{M=0}\bar{I^{M=1}}\right)P\left(I^{M=0}\bar{I^{M=1}}\right)-P\left(D^{M=0}|I^{M=0}\bar{I^{M=1}}\right)P\left(I^{M=0}\bar{I^{M=1}}\right)\left.+P\left(D^{M=1}|I^{M=0}I^{M=1}\right)P\left(I^{M=0}I^{M=1}\right)-P\left(D^{M=0}|I^{M=0}I^{M=1}\right)P\left(I^{M=0}I^{M=1}\right)\right)$$

**Fig. 4:**
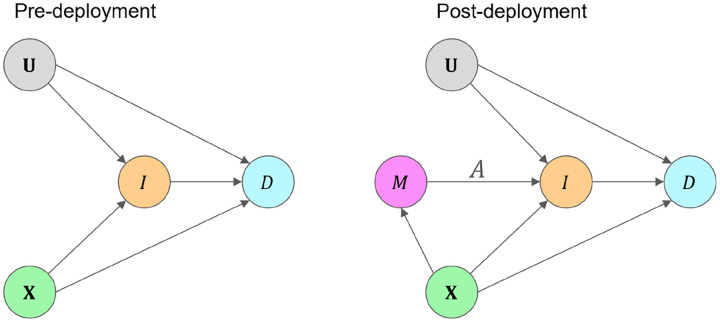
The Directed acyclic graph (DAG) of the model, resulting intervention and the outcome preand post-deployment. X are variables that impact both the clinician's decision and are part of the model input. U are variables that are only observable by the clinicians but are not included in model inputs.

The first line corresponds to patients who do not receive an intervention with or without the model. Assuming the entire causal effect of the model on the outcome passes through the intervention (Figure 4), the CE(M) for this subset is 0. Similarly, the last line focuses on patients who receive an intervention with or without the model and therefore it is 0. The second line subsets on patients who would receive an intervention in the presence of the model but not otherwise. Since the effect of the model on intervention is conditioned away, the CE is equal to the CE of the intervention on the outcome in this subset. To the contrary, the third line subsets on patients who received an intervention in the absence of the model but not in its presence. Therefore, the CE in this subset is equal to the inverse of the CE of the intervention. Hence,

(8) $$CE\left(M\right)=\left(P\left(D^{I=1}|\bar{I^{M=0}}I^{M=1}\right)-P\left(D^{I=0}|\bar{I^{M=0}}I^{M=1}\right)\right)P\left(\bar{I^{M=0}}I^{M=1}\right)+\left(P\left(D^{I=0}|I^{M=0}\bar{I^{M=1}}\right)-P\left(D^{I=1}|I^{M=0}\bar{I^{M=1}}\right)\right)P\left(I^{M=0}\bar{I^{M=1}}\right)$$

We can also write

(9) $$P\left(\bar{I^{M=0}}I^{M=1}\right)=P\left(\bar{I^{M=0}}|I^{M=1}\right)P\left(I^{M=1}\right)=P\left(I^{M=1}\right)-P\left(I^{M=0}I^{M=1}\right)$$

and

(10) $$P\left(I^{M=0}\bar{I^{M=1}}\right)=P\left(\bar{I^{M=1}}|I^{M=0}\right)P\left(I^{M=0}\right)=P\left(I^{M=0}\right)-P\left(I^{M=0}I^{M=1}\right)$$

Therefore,

(11) $$CE\left(M\right)=\left(P\left(D^{I=1}|\bar{I^{M=0}}I^{M=1}\right)-P\left(D^{I=0}|\bar{I^{M=0}}I^{M=1}\right)\right)\left(P\left(I^{M=1}\right)-P\left(I^{M=0}I^{M=1}\right)\right)+\left(P\left(D^{I=0}|I^{M=0}\bar{I^{M=1}}\right)-P\left(D^{I=1}|I^{M=0}\bar{I^{M=1}}\right)\right)\left(P\left(I^{M=0}\right)-P\left(I^{M=0}I^{M=1}\right)\right)$$

Using the Bayes’ Rule and equation 9, we can write

(12) $$P\left(D^{I=1}|\bar{I^{M=0}}I^{M=1}\right)=\frac{P\left(\bar{I^{M=0}}I^{M=1}|D^{I=1}\right)P\left(D^{I=1}\right)}{P\left(\bar{I^{M=0}}I^{M=1}\right)}\;=\frac{\left(P\left(I^{M=1}|D^{I=1}\right)-P\left(I^{M=0}I^{M=1}|D^{I=1}\right)\right)P\left(D^{I=1}\right)}{P\left(I^{M=1}\right)-P\left(I^{M=0}I^{M=1}\right)}$$

Similar identities can be derived for the conditional terms in equation 11, leading to the following results

(13) $$CE\left(M\right)=\left[P\left(I^{M=1}|D^{I=1}\right)-P\left(I^{M=0}I^{M=1}|D^{I=1}\right)\right]P\left(D^{I=1}\right)-\left[P\left(I^{M=1}|D^{I=0}\right)-P\left(I^{M=0}I^{M=1}|D^{I=0}\right)\right]P\left(D^{I=0}\right)+\left[P\left(I^{M=0}|D^{I=0}\right)-P\left(I^{M=0}I^{M=1}|D^{I=0}\right)\right]P\left(D^{I=0}\right)-\left[P\left(I^{M=0}|D^{I=1}\right)-P\left(I^{M=0}I^{M=1}|D^{I=1}\right)\right]P\left(D^{I=1}\right)$$

The terms involving the joint probabilities of intervention with and without the model cancel out and CE(M) can be calculated using only the marginal probabilities,

(14) $$CE\left(M\right)=\left(P\left(I^{M=1}|D^{I=1}\right)-P\left(I^{M=0}|D^{I=1}\right)\right)P\left(D^{I=1}\right)-\left(P\left(I^{M=1}|D^{I=0}\right)-P\left(I^{M=0}|D^{I=0}\right)\right)P\left(D^{I=0}\right)=\left(P\left(D^{I=1}|I^{M=1}\right)-P\left(D^{I=0}|I^{M=1}\right)\right)P\left(I^{M=1}\right)-\left(P\left(D^{I=1}|I^{M=0}\right)-P\left(D^{I=0}|I^{M=0}\right)\right)P\left(I^{M=0}\right)$$

When the performance of the model is evaluated post-deployment, it is often compared to the performance of the model pre-deployment since the only true measurements of performance that are ever available are from before the model is deployed, and the decisions to deploy the model are made based on pre-deployment performance. Hence, the potential effect of the model on the intervention:

(15) $$CE\left(M\right)=\left(P\left(D^{I=1}|I_{\text{Post}}\right)-P\left(D^{I=0}|I_{\text{Post}}\right)\right)P\left(I_{\text{Post}}\right)-\left(P\left(D^{I=1}|I_{\text{Pre}}\right)-P\left(D^{I=0}|I_{\text{Pre}}\right)\right)P\left(I_{\text{Pre}}\right)=CE\left(I|I_{\text{Post}}\right)P\left(I_{\text{Post}}\right)-CE\left(I|I_{\text{Pre}}\right)P\left(I_{\text{Pre}}\right)$$

where $CE\left(I|I_{\text{Pre}}\right)$ and $CE\left(I|I_{\text{Post}}\right)$ are the causal effect of treatment (intervention) on the outcome among the patients treated pre- and post-deployment, respectively.

#### 4.1.1 Estimation of Sensitivity, Specificity and PPV

Using the equations above, the PPV can be estimated as

(16) $$P\left(D^{M=0}|A\right)=\frac{P(D)-CE(M)-P(D|\bar{A})P(\bar{A})}{P(A)}=P(D|A)-\frac{CE(M)}{P(A)}$$

Similarly, sensitivity and specificity can be estimated as

(17) $$P\left(A|D^{M=0}\right)=1-\frac{P(D|\bar{A})P(\bar{A})}{P(D)-CE(M)}$$

and

(18) $$P\left(\bar{A}|\bar{D^{M=0}}\right)=\frac{P(\bar{A})-P(D|\bar{A})P(\bar{A})}{1-P\left(D^{M=0}\right)}=\frac{P(\bar{A})-P(D|\bar{A})P(\bar{A})}{1-P(D)+CE(M)}$$

The estimation equations for various performance estimates are shown in Table 2.

The CE(M) can be estimated using the causal effect of intervention on the adverse outcome for patients who received the intervention before and after deployment,

(19) $$\hat{CE}(M)=\hat{CE}\left(I|I_{\text{Post}}\right)P\left(I_{\text{Post}}\right)-\hat{CE}\left(I|I_{\text{Pre}}\right)P\left(I_{\text{Pre}}\right)$$

where $P\left(I_{\text{Pre}}\right)$ and $P\left(I_{\text{Post}}\right)$ are the observed rates of intervention pre- and post-deployment, respectively. This estimation requires two models to estimate the causal effects which can be done using a variety of different methods including approaches that use inverse probability of treatment weighting. In many scenarios, it can be assumed that the causal effect of the intervention on the outcome is not impacted by the deployment of the model; hence, one only needs to estimate CE(I∣X) predeployment, where X is the set of patient covariates, to estimate $CE\left(I|I_{\text{Post}}\right)$ and $CE\left(I|I_{\text{Pre}}\right).X$ is identified using a causal directed acyclic graph (DAG). DAGs are graphical representations of the joint probability distribution between variables in a data generating process (e.g., determinants of treatments and outcomes in a clinical environment). They are used to isolate causal relationships by identifying adjustment sets sufficient to eliminate confounding.

#### 4.1.2 Estimation of AUROC, AUPR and Calibration Plot

The ROC and PR curves are generated by measuring sensitivity, specificity and PPV at different binary thresholds. The only terms in Equations 16, 17 and 18 that depend on these thresholds are the probabilities conditioned on A or $\bar{A}$. These probabilities can be easily calculated by subsetting patients based on the prediction scores and the threshold value, A(τ) where τ is the classifier threshold. The sensitivity, specificity and PPV are then calculated for each subsets by substituting A(τ) in Equations 16, 17 and 18. The results are used to construct the ROC and PR curves and the AUCs are calculated using Trapezoidal integration.

The calibration plot shows the relationship between the model’s predicted probability of the event and the true probability in the absence of the AI-DST. Similar to other performance metrics, the observed probability is biased and does not reflect the true pre-deployment probability of the event. However, the true pre-deployment probability of event can easily be calculated as P(D)-CE(M) to construct the calibration plot. The results of the calibration plot estimation are not shown in this work for brevity.

### 4.2 Performance Estimation when AI-DST is not the Only Source of CMI

In most clinical settings, it is not reasonable to assume that the there are no other changes to the system that impact the clinician decisions or the model outcomes after the deployment of the AI-DST (Scenario C in Figure 1). For the more general case depicted as Scenario D in Figure 1, we allow other new biomarkers (e.g., new lab tests or another predictive model) to impact $P\left(I_{\text{Post}}\right)$. Imagine a new model, $M^{\prime }$, which is a combination of M and the new biomarkers. Since the observed interventions are impacted by both the AI-DST and the new biomarkers, $P\left(D^{M=1}\right),CE(M)$ and $P(\bar{A})$ cannot be estimated from the observed data anymore. However, we can use the fact that $P\left(D^{M=1}\right)-CE(M)=P\left(D^{M=0}\right)$ and $P\left(D^{M^{\prime }=1}\right)-CE\left(M^{\prime }\right)=P\left(D^{M^{\prime }=0}\right)$. Since both M=0 and $M^{\prime }=0$ correspond to the pre-deployment setting, we can conclude that $P\left(D^{M=1}\right)-CE(M)=P\left(D^{M^{\prime }=1}\right)-CE\left(M^{\prime }\right)$. As a result, the contrast $P\left(D^{M=1}\right)-CE(M)$ is not impacted by the new biomarkers. Hence, we can rewrite the equation for PPV as

(20) $$P\left(D^{M=0}|A\right)=\frac{P\left(D^{M=1}\right)-CE\left(M\right)-P\left(D^{M=1}|\bar{A}\right)P\left(\bar{A}\right)}{P\left(A\right)}\;=\frac{P\left(D^{M^{\prime }=1}\right)-CE\left(M^{\prime }\right)-P\left(D^{M=1}|\bar{A}\right)P\left(\bar{A}\right)}{P\left(A\right)}$$

The other term in the PPV equation that is impacted by the presence of the new biomarkers is $P\left(D^{M=1}|\bar{A}\right)$. However, under the assumption that the model impacts interventions and outcomes through the alerts A, the probability of adverse outcome pre- and post-deployment are equal for patients who are not predicted as positive, hence $P\left(D^{M=1}|\bar{A}\right)=P\left(D^{M=0}|\bar{A}\right)$. As a result, we can accurately calculate the model PPV in the presence of new biomarkers that impact P(I) by replacing $P\left(D^{M=1}|\bar{A}\right)$ with $P\left(D^{M=0}|\bar{A}\right)$ which can be calculated from the retrospective pre-deployment data. Hence, the PPV in Scenario D can be estimated using observable quantities:

(21) $$P\left(D^{M=0}|A\right)=\frac{P\left(D^{M^{\prime }=1}\right)-CE\left(M^{\prime }\right)-P\left(D^{M=0}|\bar{A}\right)P(\bar{A})}{P(A)}$$

Similarly, sensitivity and specificity can be calculated by replacing $P\left(D^{M=1}\right)-CE(M)$ with $P\left(D^{M^{\prime }=1}\right)-CE\left(M^{\prime }\right)$ and $P\left(D^{M=1}|\bar{A}\right)$ with $P\left(D^{M=0}|\bar{A}\right)$.

### 4.3 Estimation Bias due to Unmeasured Confounding

Accurate treatment effect estimates will require multiple untestable assumptions. Most importantly, unmeasured confounding can lead to bias in the estimated CE(I). The pre-deployment bias, $b_{\text{Pre}}$, can be formulated as

(22) $$b_{\text{Pre}}=E\left[\hat{CE}\left(I|I_{\text{Pre}}\right)\right]-CE\left(I|I_{\text{Pre}}\right)\;\Rightarrow E\left[\hat{CE}\left(I|I_{\text{Pre}}\right)\right]=b_{\text{Pre}}+CE\left(I|I_{\text{Pre}}\right)$$

Similarly, we can derive $E\left[\hat{CE}\left(I|I_{\text{Post}}\right)\right]=b_{\text{Post}}+CE\left(I|I_{\text{Post}}\right)$. Therefore, the bias in the estimated causal effect of the model can be calculated as

(23) $$E\left[\hat{CE}\left(M\right)\right]-CE\left(M\right)=E\left[\hat{CE}\left(I|I_{\text{Post}}\right)\right]P\left(I_{\text{Post}}\right)-E\left[\hat{CE}\left(I|I_{\text{Pre}}\right)\right]P\left(I_{\text{Pre}}\right)-CE\left(I|I_{\text{Post}}\right)P\left(I_{\text{Post}}\right)+CE\left(I|I_{\text{Pre}}\right)P\left(I_{\text{Pre}}\right)=\left(b_{\text{Post}}+CE\left(I|I_{\text{Post}}\right)\right)P\left(I_{\text{Post}}\right)-\left(b_{\text{Pre}}+CE\left(I|I_{\text{Pre}}\right)\right)P\left(I_{\text{Pre}}\right)-CE\left(I|I_{\text{Post}}\right)P\left(I_{\text{Post}}\right)+CE\left(I|I_{\text{Pre}}\right)P\left(I_{\text{Pre}}\right)=b_{\text{Post}}P\left(I_{\text{Post}}\right)-b_{\text{Pre}}P\left(I_{\text{Pre}}\right)$$

Therefore, the bias in the estimated PPV is equal to

(24) $$E\left[\hat{P}\left(D^{M=0}|A\right)\right]-P\left(D^{M=0}|A\right)=P\left(D|A\right)-\frac{E\left[\hat{CE}\left(M\right)\right]}{P\left(A\right)}-\left(P\left(D|A\right)-\frac{CE\left(M\right)}{P\left(A\right)}\right)\;=\frac{b_{\text{Post}}P\left(I_{\text{Post}}\right)-b_{\text{Pre}}P\left(I_{\text{Pre}}\right)}{P\left(A\right)}$$

In many scenarios, the unmeasured confounding is not impacted by the model deployment. Hence, $b_{\text{Post}}=b_{\text{Pre}}=b$ and the bias simplifies to

(25) $$E\left[\overset{ˆ}{P}\left(D^{M=0}|A\right)\right]-P\left(D^{M=0}|A\right)=b\frac{P\left(I_{\text{Post}}\right)-P\left(I_{\text{Pre}}\right)}{P(A)}$$

Therefore, the bias in PPV estimates scales with the ratio of the number of additional interventions that are introduced by the model to the number of alerts that are generated by the model. If the intervention is resource constrained such as ICU transfer in a setting where the ICU is often at capacity, a predictive model cannot increase the intervention rate. Instead, it would impact which patients will be transferred to the ICU and hence $P\left(I_{\text{Post}}\right)-P\left(I_{\text{Pre}}\right)\approx 0$. Therefore, the bias in the PPV estimate will be approximately 0 and will be minimally impacted by unmeasured confounding.

### 4.4 Simulations

To test the equations that were derived in the previous sections, a simulation study was developed where the intervention probability for patient p being treated by clinician c was calculated as

(26) $$P\left(I_{p}\right)=\sigma \left(\gamma_{c}^{\text{all}}x_{p}^{\text{all}}+\gamma_{c}^{\text{clinician}}x_{p}^{\text{clinician}}+\gamma_{c}^{\text{alert}}u_{p}a_{p}+\gamma_{c}^{\sim \text{alert}}u_{c}\left(1-a_{p}\right)+\gamma_{c}^{\text{promoter}}x_{p}^{\text{promoter}}+\gamma_{c}^{0}\right)$$

where σ is the sigmoid function and $\gamma_{c}^{\text{all}},\gamma_{c}^{\text{clinician}},\gamma_{c}^{\text{alert}},\gamma_{c}^{\sim \text{alert}},\gamma_{c}^{\text{promoter}}$ and $\gamma_{c}^{0}$, represent how much clinician c weighs factors that are part of the AI-DSTs input variables (observable to all), factors that are only available to the clinician, the AI-DST alerts, the absence of AI-DST alerts, another promoter of the same intervention that the model targets, and the baseline level of intervention, respectively. In addition, $x_{p}^{\text{all}},x_{p}^{\text{clinician}},x_{p}^{\text{promoter}}$ represent the patient covariates that are available to both the model and clinician, the covariates that are only available to the clinician, and another factor (another model, biomarker, clinical guideline, etc.) that impacts the rate of the same intervention as the one the AI-DST targets and was introduced after the AI-DST was tested. $a_{p}$ is a binary variable indicating whether the patient had a positive prediction and $u_{p}$ represents whether the AI-DST alert was used for patient p.

The probability that patient p experiences the adverse outcome that is the target of the predictive AI-DST was calculated as

(27) $$P\left(D_{p}\right)=\sigma \left(\alpha_{p}^{\text{all}}x_{p}^{\text{all}}+\alpha_{p}^{\text{clinician}}x_{p}^{\text{clinician}}+\alpha_{p}^{\text{unobserved}}x_{p}^{\text{unobserved}}+\alpha_{p}^{\text{intervention}}I_{p}e_{p}+\alpha_{p}^{0}\right)$$

where $\alpha_{p}^{\text{all}},\alpha_{p}^{\text{clinician}},\alpha_{p}^{\text{unobserved}},\alpha_{p}^{\text{intervention}}$ and $\alpha_{p}^{0}$ represent the contributions of the factors that are observable to both the model and the clinician, factors that are only observable to the clinician, factors that are not observable to either the model or the clinician, whether the patient received an intervention, and the baseline propensity to experience the event, to the probability that the patient experiences the adverse outcome. In addition, $x_{p}^{\text{unobserved}}$ corresponds to the patient covariates that are not observed by either the model or the clinician, and $e_{p}$ corresponds to the effectiveness of the intervention on patient p.

To test the proposed methods, 100 clinicians were simulated. $\lambda_{c}\sim N(0.8,0.1)$ represented the probability that the clinician uses the AI-DST in their decision making. In addition, 1,000,000 patients were simulated and one clinician was randomly assigned to each patient. The patient covariates were simulated as $x_{p}^{\text{all}}\sim \text{Exp}(0.2),x_{p}^{\text{clinician}}\sim \text{Exp}(0.1),x_{p}^{\text{unobserved}}\sim \text{Exp}(0.25),x_{p}^{\text{promoter}}\sim N(1,1)$ and $e_{p}\sim \text{Exp}(1)$. In addition, $u_{p}\sim \text{Bernoulli}\left(\mu_{c}\right)$ where $\mu_{c}$ is the propensity of clinician c who is treating the patient to use the AI-DSTs alerts and is distributed as N(0.65,0.1). We define the AI-DST to be a direct function of $x_{p}^{\text{all}}$, i.e., $s_{p}=\sigma \left(x_{p}^{\text{all}}\right)$ where $s_{p}$ is patient p’s predicted scores. The simulation were run a 100 times, varying $\gamma_{c}^{\text{alert}}$ from 0.1 to 5.4 on a log scale to simulate different levels of a intervention. The values of the remaining parameters in each scenario are shown in Table 4.

## Figures and Tables

**Table 1:** Comparison of the proposed work against existing solutions to monitor AI-DSTs in the presence of CMIs. Green, yellow and red denote full, partial and no support, respectively.

Approach	Scenarios (see Figure 1)	Handles Non-Causal AI-DSTs	Supported Performance Metrics	Direct Performance Estimation	Applications
Feng et al.13	A	Yes	Calibration	No	Monitoring, Retraining
Feng et al.14	A	Yes	Sensitivity, PPV	No	Monitoring
Lenert et al.15	A-D	No	None	Yes	Monitoring
**This work**	**A-D**	**Yes**	**Sensitivity, Specificity, PPV, NPV, Calibration**	**Yes**	**Monitoring, Retraining**

**Table 2:** Summary of derived equations for model performance metrics using observed quantified and CE(M).

Metric	Estimation
Sensitivity	$1-\frac{P(D|\bar{A})P(\bar{A})}{P(D)-CE(M)}$
Specificity	$\frac{P(\bar{A})-P(D|\bar{A})P(\bar{A})}{1-P(D)+CE(M)}$
PPV	$P(D|A)-\frac{CE(M)}{P(A)}$
NPV	$1-P(D|\bar{A})$

**Table 3:** Notations used throughout this paper.

Variable	Definition
D,I,A	Outcome, intervention and alert (positive prediction)
$D^{M=0},D^{M=1}$	Potential outcome in the absence and presence of model
$I^{M=0},I^{M=1}$	Potential intervention in the absence and presence of model
$I_{\text{Pre}},I_{\text{Post}}$	Patients who are treated pre- and post-deployment
CE(M),CE(I)	Causal effect of model and intervention on outcome
$CE\left(I|I_{\text{Pre}}\right)CE\left(I|I_{\text{Post}}\right)$	Causal effect of the intervention on outcome among the treated patients pre- and post-deployment

**Table 4:** The simulation parameters.

Parameter	Scenario A	Scenario B	Scenario C	Scenario D
$\gamma_{c}^{\text{all}}$	0	1	1	1
$\gamma_{c}^{\text{clinician}}$	0	0	0	0
$\gamma_{c}^{\text{alert}}$	variable	variable	variable	variable
$\gamma_{c}^{\sim \text{alert}}$	0	0	$-\gamma_{c}^{\text{alert}}$	$-\gamma_{c}^{\text{alert}}$
$\gamma_{c}^{\text{promoter}}$	0	0	0	1
$\gamma_{c}^{0}$	0	0	0	0
$\alpha_{p}^{\text{all}}$	1.5	1.5	1.5	1.5
$\alpha_{p}^{\text{clinician}}$	1	1	1	1
$\alpha_{p}^{\text{unobserved}}$	1	1	1	1
$\alpha_{p}^{\text{intervention}}$	−10	−10	−10	−10
$\alpha_{p}^{0}$	−2	−2	−2	−2

## Data Availability

The data supporting the findings presented in this paper are available to researchers upon reasonable request. To request data, please email the corresponding author.

## Code availability

The codes used to generate the results presented in this publication are available on reasonable request. Please email the corresponding author for access.
